# Asgard archaea are the closest prokaryotic relatives of eukaryotes

**DOI:** 10.1371/journal.pgen.1007080

**Published:** 2018-03-29

**Authors:** Anja Spang, Laura Eme, Jimmy H. Saw, Eva F. Caceres, Katarzyna Zaremba-Niedzwiedzka, Jonathan Lombard, Lionel Guy, Thijs J. G. Ettema

**Affiliations:** 1 Department of Cell and Molecular Biology, Science for Life Laboratory, Uppsala University, Uppsala, Sweden; 2 Department of Microbiology, Oregon State University, Corvallis, Oregon, United States of America; 3 Department of Medical Biochemistry and Microbiology, Uppsala University, Uppsala, Sweden; Vanderbilt University, UNITED STATES

In Spang et al. [1], we reported the discovery of Lokiarchaeum (Loki1) and two related lineages (Loki2 and Loki3, now referred to as Heimdallarchaeote LC_2 and LC_3). These were the first known representatives of the subsequently described Asgard superphylum [2], which branches as a sister clade to the TACK archaea [3]. We provided extensive phylogenomic evidence for the emergence of eukaryotes from within the Asgard archaea (thus supporting the two-domains [2D] tree of life), and the investigation of the Lokiarchaeum genome revealed the presence of more eukaryotic signature proteins (ESPs) than in any other described archaeal lineage [1].

In the recent study by Da Cunha et al. [4], the authors argue against the findings reported in Spang et al. Their main claims are that (i) the published genome data contains extensive contamination from distantly related organisms; (ii) a subset of universal genes supports a three-domains (3D) tree of life; and (iii) removing the elongation factor 2 (EF2) from the phylogenomic analysis breaks the Lokiarchaeota–Eukarya affiliation.

Below, we show that the claims by Da Cunha et al. are unfounded.

## There is no evidence of significant contamination in the lokiarchaeal genomes

Da Cunha et al. determined the quality of the Loki1 genome bin with CheckM [5] and observed 78.21% heterogeneity, which they incorrectly interpreted as a sign of contamination. CheckM measures heterogeneity based on the detection of multiple copies of single-copy marker genes, and in fact, 78.21% of the redundant markers share at least 90% amino acid identity. This confirms our original report, which reported that the Lokiarchaeum genome bin comprises sequences from closely related strains [1].

The authors argue that single-gene trees indicate contamination because Asgard homologs are not always monophyletic. However, given that Loki1–3 representatives belong to the distinct phyla Lokiarchaeota and Heimdallarchaeota, this is not surprising [2]. Furthermore, most deep nodes in these single-gene trees are not significantly supported, which hinders reaching strong conclusions regarding the placement of these sequences.

Da Cunha et al. also state that the single gene–encoding RNA polymerase subunit A (*rpoA*) represents a contaminant of the Heimdallarchaeote LC_3 bin, because *rpoA* is encoded by two genes in other Asgard archaea, while some lineages within TACK encode a single gene [2]. BLAST searches for the N- and C-termini of the Heimdallarchaeote LC_3 *rpoA* gene sequence actually recover best hits to other Asgard archaea, thus indicating that it represents a bona fide Heimdallarchaeote gene. In addition, Heimdallarchaeote LC_3 RpoA forms a strongly supported monophyletic group with the homologs of all other Asgard archaea in phylogenetic analyses [2]. Finally, split/fusion events of archaeal RpoA subunits appear to have occurred more frequently than assumed previously [2], rendering them unsuited for the elucidation of distant evolutionary events.

Moreover, the authors claim that lokiarchaeal EF-2 homologs contain indels indicative of chimerism. However, it is unlikely that chimerism has affected the same gene in all Loki- and Heimdallarchaeota independently while leaving all other genes in these genomes unaffected. The evolution of the EF-2 homologs in archaea is actually a much more complex subject than previously acknowledged. An analysis of the archaeal EF-2 paralogs and their characteristics is already available in [2] and will be discussed in greater detail in a devoted article (Narrowe et al., submitted).

Finally, most of the work by Da Cunha et al. limits itself to the first lokiarchaeal genome bin [1] and disregards the eight additional metagenomic bins from a diversity of Asgard archaea [2]. The currently available Asgard genomes stem from five different samples and metagenomes that were assembled and binned by three independent laboratories using different methodologies. All of them corroborate our original phylogenetic placement of Asgard lineages, their relationship with eukaryotes, and the unique presence of ESPs in all of these genomes.

## The 3D topology recovered in a subset of universal genes is likely due to long-branch attraction artefacts

After running topology tests, Da Cunha et al. argue that only 6 and 11 universal markers statistically rejected 2D and 3D topologies, respectively. They split these two datasets into respective concatenations and reconstructed the corresponding phylogenies (S7 and S8 Figs in [4]). Unsurprisingly, they found a 3D topology for the 6-protein dataset, and a 2D topology, where eukaryotes and Asgard archaea are monophyletic, for the 11-protein concatenation. As the number of individual trees showing a 2D topology is higher when additional longer-branching taxa are included (S1 Fig [4]), the authors incorrectly argue that the topology obtained by the 11-protein dataset is likely the result of a long-branch attraction (LBA) artefact. By definition, LBA artefactually attracts long-branching lineages together, irrespective of their correct phylogenetic placement. Thus, if LBA was affecting the phylogenetic reconstruction, the eukaryotic clade would be attracted outside the archaea, to the distantly related bacterial outgroup, and not well nested within the archaeal clade. Rather, we argue that the 3D topology obtained by Da Cunha et al. for the 6-protein dataset is itself the result of LBA: the branch length connecting the bacterial outgroup to the archaeal and eukaryotic clade is strikingly longer in the 6-protein phylogeny compared to the 11-protein tree (2.1 versus 1.3 average substitution per site). The existence of artefacts in the 3D topology is further supported by the fact that the internal relationships among eukaryotes are less consistent with the accepted phylogeny of organisms than in the 2D tree. For example, the fast-evolving *Entamoeba histolytica* is attracted toward the base in the 3D tree, whereas it is correctly placed as sister to *Dictyostelium discoideum* in the 2D tree. The 3D topology reported in Da Cunha et al. is thus most likely incorrect.

Finally, it is important to acknowledge that the 2D topology has been obtained by numerous studies worldwide using different datasets and methods [3,6–14]; several of these studies were published before the publication of the Lokiarchaeum genome.

## Phylogenetic analyses excluding EF2 support the monophyly of Asgards and eukaryotes

The claim “removal of the EF-2, is sufficient to break the Eukaryotes-Lokiarchaea affiliation” [4] has been disproven by phylogenetic analyses performed on three different concatenated datasets [2], none of which included elongation factor EF-2 (48 universal single-copy genes, 55 ribosomal proteins, and 16S and 23S rRNA genes, respectively). All analyses clearly retrieved a strongly supported affiliation of eukaryotes with the Asgard superphylum.

## Conclusions

The origin of eukaryotes represents a controversial topic, and while we believe it to be refreshing and insightful, our recent work will certainly not be the final word in this debate. However, Da Cunha et al. use inadequate methodology, misinterpret data and ignore previous work [1,2] that already addressed many of their criticisms. In summary, they provide no evidence to falsify the conclusions drawn in Spang et al. and Zaremba-Niedzwiedzka et al.
